# Anatomical Characterization, HPLC Analysis, and Biological Activities of *Ilex dipyrena*

**DOI:** 10.3390/plants11050617

**Published:** 2022-02-24

**Authors:** Amjad Ali, Mohammad Nisar, Syed Wadood Ali Shah, Atif Ali Khan Khalil, Muhammad Zahoor, Nausheen Nazir, Sayed Afzal Shah, Fahd A. Nasr, Omar M. Noman, Ramzi A. Mothana, Sajjad Ahmad, Hafiz Muhammad Umer Farooqi

**Affiliations:** 1 Department of Botany, University of Malakand, Dir (Lower), Chakdara 18800, Khyber Pakhtunkhwa, Pakistan; amjad1990.aa48@gmail.com; 2 Department of Pharmacy, University of Malakand, Dir (Lower), Chakdara 18800, Khyber Pakhtunkhwa, Pakistan; pharmacistsyed@gmail.com; 3 Department of Biological Sciences, National University of Medical Sciences, Rawalpindi 46000, Punjab, Pakistan; atif.ali@numspak.edu.pk (A.A.K.K.); afzal.shah@numspak.edu.pk (S.A.S.); 4 Department of Biochemistry, University of Malakand, Dir (Lower), Chakdara 18800, Khyber Pakhtunkhwa, Pakistan; farhatkhan2k9@yahoo.com (M.Z.); nausheen.nazir@uom.edu.pk (N.N.); 5 Department of Pharmacognosy, College of Pharmacy, King Saud University, Riyadh 11451, Saudi Arabia; fnasr@ksu.edu.sa (F.A.N.); onoman@ksu.edu.sa (O.M.N.); rmothana@ksu.edu.sa (R.A.M.); 6 Department of Pharmacy, Sarhad University of Information Technology, Peshawar 25000, Khyber Pakhtunkhwa, Pakistan; sajad4u2000@yahoo.com; 7 Department of Mechatronics Engineering, Jeju National University, Jeju-si 63243, Korea; umerfarooqi@jejunu.ac.kr (H.M.U.F.)

**Keywords:** anatomy, microscopy, antioxidant, anti-inflammatory, *Ilex dipyrena*

## Abstract

*Ilex dipyrena* Wall (*Aquifoliaceae*), is a traditional medicinal plant abundantly found in India and Pakistan. In the current research work, initially, the anatomical characteristics were recorded through microscopic examination of selected plant parts, such as leaf, petiole, and midrib. Then, the quantitative phytochemical screening was performed using standard tests reported in literature. The whole-plant powdered sample was then soaked in methanol to obtain crude extract, which was then fractionated into solvents of different polarities to obtain ethyl acetate, chloroform, butanol, hexane, and aqueous extracts. The phytochemical composition of the crude ethyl acetate and chloroform extracts (being the most active fractions) was then confirmed through HPLC analyses, where the possible phytochemical present were predicted through comparison of retention time of a given compound peak with the available standards. The extracts were also evaluated for their in vitro antioxidant and ani-lipoxygenase potentials using standard methods. The microscopic examination revealed the presence of anomocytic type stomata on the abaxial side of the leaf as well as unicellular trichrome and calcium oxalate druses crystals in the midrib and petiole, with a single, centered U-shaped collateral arterial bundle, which was directed toward the adaxial and the phloem toward the abaxial sides of the selected plant parts, respectively. Almost all tested representative groups of phytochemicals and essential minerals were detected in the selected plant, whereas five possible phytochemicals were confirmed in crude and chloroform extract and seven in ethyl acetate fraction. As antioxidant, chloroform fraction was more potent, which exhibited an IC_50_ value of 64.99, 69.15, and 268.52 µg/mL, determined through DPPH, ABTS, and FRAP assays. Ethyl acetate extract was also equally potent against the tested free radicals. Chloroform and ethyl acetate extracts were also potent against lipoxygenase, with IC_50_ value of 75.99 and 106.11 µg/mL, respectively. Based on the results of biological studies, *Ilex dipyrena* was found to good inhibitor of free radicals and lipoxygenase that could be further investigated to isolate compounds of medicinal importance.

## 1. Introduction

Plants as factories of natural products are constantly investigated by human beings since the beginning of human history on earth. As living organisms, plants can also be infected by microbes and need medication [1]. However, nature has made them capable of synthesizing secondary metabolites, the phytochemicals of defense and offense, which enable plants to protect themselves from diseases [2]. The prehistoric human utilized this simple logic, which eventually led to the foundation of modern pharmacopoeia. Today, plants are investigated for their various biological potentials with modern, sophisticated machines, and more than 90% of the drug industry is totally or in-part dependent on plant products [3]. Although tremendous advancements have been made in medicinal sciences since 1950, at the same time, pathogens have also adapted themselves to cope with the newly developed drugs, giving rise to the problem of drug resistance. Scientists are trying to introduce new drugs capable of combating infections with new mechanism [4]. That is the why the research on plants has tremendously increased from last few decades. Reactive oxygen species are normally produced during respiration and are even required for oxidative bursts while killing the microbes with white blood cells. However, their increased concentration can lead to a number of health complications, ranging from aging to life-threatening diabetes and cancer [2,3]. In plants, mostly flavonoids/phenolics are excellent scavengers of these radicals, and that is the reason that physicians stress the use of fresh vegetables and fruits [4]. Lipoxygenase has a key role in inflammation as a mechanism of expression of infection in a particular part of the body to attract monocytes and an adaptive immune component at the site of infection to combat the infection. Plant-based inhibitors are preferably used to inhibit these enzymes and relieve the severity of the inflammation [5]. 

*Ilex* (*Aquifoliaceae*) is a family of about 400 species that grows in tropical and temperate regions of the world. In India, there are around 24 kinds of evergreen deciduous trees and plants belonging to this family [6]. Almost all plants of this family contain all the representative phytochemical groups, such as saponins [7], flavonoids [8], aldehydes [9], hemiterpene glycosides, triterpenes [10], etc. That is why majority of the species of this family are utilized as remedies of various diseases in various parts of the world. *Ilex latifolia* extracts have previously been shown to have significant anti-inflammatory and antinociceptive properties in both in vitro and in vivo models of inflammation and pain [11]. In *I. pubescens* root, saponin fractions were purified and tested in both visceral and cerebral nociceptive models, and they demonstrated a strong analgesic effects [12]. Similarly, dried *I. paraguariensis* leaves and twigs (yerba mate) are used in the preparation of a local tea known in some parts of the world as mate, which is a popular beverage in many South American countries. Theobromine and caffeine, which are alkaloids with medicinal and pharmacological properties, have been identified in *I. paraguariensis* [13]. These substances have been found to have effects on the central nervous system [13,14].

**Figure 1:**
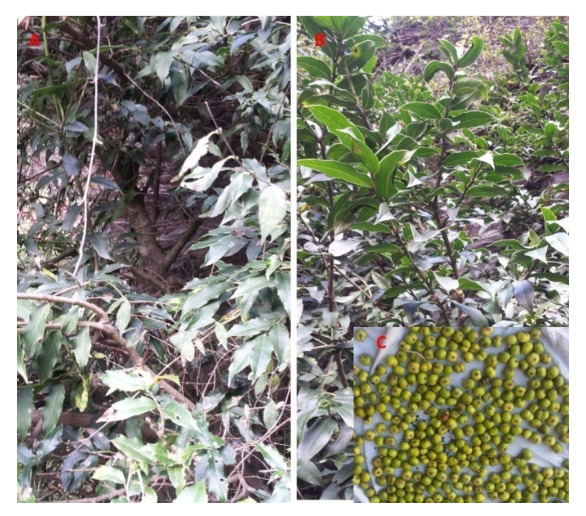
*I. dipyrena* plant and its various parts collected from Shangla, Khyber-Pakhtunkhwa, Pakistan.

*Ilex dipyrena* Wall (Figure 1), a member of the Aquifoliaceae family, is an evergreen tree that reaches a height of about 10 m [12]. It can reach to a height of 2 to 15 m normally; however, in some cases, it can reach a 25-m height. *I. dipyrena* has been investigated for its phytoconstituents through GC-MS, and a number of fatty acids have been reported from it [15]. Another GC-MS analysis of leaf, bark, and fruit extracts of the same plant have shown the presence of cathinone, phenylpropanolamine, dl-phenylephrine, amphetamine, myristic acid, and palmitic acid, etc. [16]. The crude ethanolic extract of *Ilex dipyrena* has also been shown to have antimicrobial property [15]. Furthermore, our previous work demonstrated that the leaf, bark, and fruit extracts of this plant have analgesic and antioxidant activities [16,17]. 

In spite of the fact that we have published two papers on other aspects of the same plant [16,17], the anatomical characterization and HPLC analysis of this plant have remained unexplored. Furthermore, not enough information is available in the literature about the antioxidant and anti-inflammatory potential of this plant. Therefore, the current experimental analysis aimed to explore the anatomical phytochemicals through HPLC and biological activities, such as antioxidant and anti-inflammatory potentials, of various extracts of *Ilex dipyrena*. 

## 2. Materials and Methods

### 2.1. Collection and Authentication of Plant Material

The mature plant of *I. dipyrena* Wall. was collected from Shangla, Khyber-Pakhtunkhwa in July 2018. The plant was identified by Professor Mehboob ur Rahman, Department of Botany, Jehanzeb Postgraduate College, Swat, Pakistans. The collected specimen were properly dried in accordance with the standard conditions prescribed for preservation of plant specimen, in the herbarium University of Malakand (voucher number; BG/ID/18-244).

### 2.2. Chemicals

Methanol, chloroform, hexane, ethyl acetate, butanol, tocopherol, and indomethacin were bought from Merck (Darmstadt, Germany). HPLC-grade solvents were used in the HPLC analysis and were purchased from Daejung, Korea. The HPLC standards, ellagic acid, epigallocatechin gallate, malic acid, pyrogallol, rutin, catechin hydrate, and morin were obtained from Sigma-Aldrich, St. Quentin Fallavier, France.

### 2.3. Macroscopic Evaluations

Morphological features of bark, stem, petiole, and leaf surfaces were also recorded visually. Height in meter of the plant was assessed randomly, and the mean data of 10 plants were recorded [18]. 

### 2.4. Microscopic Evaluations

Microscopic examination of *I. dipyrena* pieces made through an automated microtome [18] were also performed. Nearly matured and healthy samples (bark, petiole, midrib, and leaf adaxial and abaxial surfaces) were taken from four plants. About 6 pieces of the *I. dipyrena* leaf, the central region of the petiole, midrib lower portion, and the bark, were collected for the measurement. The transverse sectioning was performed with a hand slicer. On slide glass, appropriately pre-arranged material was placed in 50 percent glycerinated water. For the inner morphological characteristics, an Olympus BX53 photomicroscope (Olympus, Shinjuku City, Japan) was used. On each photomicrograph, more than ten places were measured, and the mean of these measurements was selected as representative of the subject/specimen under consideration. For each plant, a minimum of three specimens was studied as suggested in literature [19]. 

### 2.5. Extraction

The whole plant (4.5 kg) in powdered form was macerated in 100% methanol, stirred occasionally (3–4 times/day) for 15 days at 25 ± 2 °C, and filtered. The filtrates were concentrated with the help of rotary evaporator, resulting in 569 g of crude extract (Crd-Id). The resulting crude extract was then suspended in water and partitioned with n-hexane (n-hex; 29 g, ethyl acetate ((Et-Id; 75 g), chloroform (Chl-Id; 63 g), and butanol (But-Id; 27 g) with residual aqueous (Aq-Id) fraction (331 g) [16]. 

### 2.6. Quantitative Phytochemical Analysis

The presence of alkaloids, flavonoids, saponins, and terpenoids in various fractions of *I. dipyrena* were determined using a non-spectrophotometric method.

#### 2.6.1. Quantification of Alkaloids

About 10 mg of each plant sample prepared in in ethanol was mixed with approximately 7.5 mL of acetic acid, resulting in a final concentration of 10% acetic acid. The sample in covered form was left for 4 h. The mixture was then filtered, and on the water bath, the resulting filtrate was concentrated to reduce its volume to one-fourth. The final step was to precipitate the extract sample by adding concentrated NH_4_OH drop by drop in the resulting mixture. Precipitates were collected on settling at the bottom. To assess the alkaloid proportion in the various plant samples studied, the precipitate obtained after washing with dilute NH_4_OH was thoroughly dried and weighed [20].

#### 2.6.2. Quantification of Flavonoids

About 100 mg of plant extract was dissolved in 80% aqueous methanol (10 mL) at room temperature, followed by filtering the extract through filter paper (Whatman-42). After being transferred into a crucible, the filtrates were subjected to complete dryness on a water bath for several hours. After gaining a constant weight, the samples were weighed [20]. 

#### 2.6.3. Quantification of Saponins

About 100 mg of each plant sample was mixed in 15 mL of aqueous ethanol, which was then evaporated to 20% of its original volume. The suspension was heated in a water bath at 55 °C for 4 h with constant shaking. The mixture was filtered and then re-extracted with another 15 mL aliquot of aqueous ethanol. Both extracts were combined and concentrated to a final concentration of 4 mL in a water bath at 90 °C. In a separating funnel, 10 mL of di-ethyl ether were added two times to the concentrated solution. Then, the aqueous layer was collected, while the ether layer was discarded. Finally, 5 mL of *n*-butanol were added to the aqueous layer, which were then washed twice with 1 mL of 5% aqueous sodium chloride. On a water bath, the solvents were evaporated till dryness, and the residual mass left was considered as saponin contents [20]. 

#### 2.6.4. Quantification of Terpenoids

About 100 mg of plant sample was soaked in alcohol and left at room temperature for 24 h. Using petroleum ether, the sample was extracted the next day. The ether extract obtained was used to determine the total amount of terpenoids present in the sample following procedure described in literature [20]. 

### 2.7. Determination of Mineral Contents

Potassium (K^+1^), calcium (Ca^+2^), iron (Fe^+3^), zinc (Zn^+2^), magnesium (Mg^+2^), and manganese (Mn^+2^) contents were determined using Atomic Absorption Spectrophotometer (Perkin Elmer AAnalyst 700 model AAS) and Flame Photometer (Elico-CL378) [21].

### 2.8. Samples Preparation for HPLC-UV Analysis

HPLC-UV analysis of extracts was carried out according to reported method in literature [22]. About 1 g of extract was combined with methanol and water mixture (1:1; 20 mL; *v*/*v*) and heated in a water bath at 70 °C for 1 h, then centrifuged for 10 min at 4000 rpm. About 2 mL of the resultant mixture was filtered and loaded to an Agilent-1260 HPLC infinity system (Santa Clara, CA, USA) equipped with an auto-sampler, quaternary pump, detector, and degasser. The Agilent-Zorbax-Eclipse column (XDB-C18; 5 µm, 4.6 × 150 mm) was used to achieve the separations. The sample was eluted through mixtures of solvent comprising of deionized water:methanol:acetic acid in different ratios as described before [22]. The spectra was recorded at 350 nm. The eluted chemicals were identified by comparing their retention time, UV spectra, and accessible standards, and the % peak area was used to quantify them.

### 2.9. Antioxidant Activity

#### 2.9.1. DPPH Free Radical Scavenging Assay

DPPH assay with a few minor modifications was used to determine the antioxidant potential of extracts [23]. To make a 0.01-mM DPPH solution, 4 mg of DPPH was dissolved in 100 mL methanol. In methanol, the test sample stock solutions (crude extract and fractions 1 mg/mL each) were prepared, and varied concentrations were obtained through the use of dilution formula. About 100 μL from each concentration was added to 3 mL of DPPH solution. The absorbance was recorded at 517 nm after 30 min of incubation in dark. Tocopherol was used a standard. Graph pad prism was used for the calculation of the IC_50_ values of each tested sample. The percentage DPPH radical scavenging potential was measured using following equation:

(1) $$\%Radical\;scavenging\;potential=\frac{C_{Abs}-S_{Abs}}{C_{Abs}}\times 100$$

The absorbance of the control sample/standard is represented by *C_Abs_*, while the absorbance of the test sample/standard is represented by *S_Abs_*.

#### 2.9.2. ABTS Scavenging Assay

Crude methanolic extract and fractions of *I. dipyrena* were also tested for their antioxidant properties against ABTS free radical. About 2.45 mM potassium persulfate and 7 mM ABTS were mixed in clean rinsed beaker and left over for 24 h in the dark. The volume of mixture was adjusted with methanol to 0.75 at 745 nm. The extract and fractions volume of 300 µL were mixed with 3 mL of ABTS solution and incubated for 6 min. The absorbance was recorded in triplicate at 745 nm, whereas tocopherol was used as standard following same procedure as described above [23].

#### 2.9.3. Determination of Ferric Reducing Power

The reducing power of extracts was determined as per following details: about 1 mL of test sample solution (10 µg/mL) was mixed with 2.5 mL of potassium ferricyanide (1% *w*/*v*) and phosphate buffer of 2.5 mL (0.2 M, pH 6.6). Then the mixture was incubated at 50 °C for 20 min, to which 2.5 mL of trichloroacetic acid (10%) were then added. After centrifugation of the mixture for 10 min at 3000 rpm, 2.5 mL were collected from the upper layer, mixed with 2.5 mL of distilled water, and 0.5 mL of FeCl_3_ (0.1%) and the absorbance was measured at 700 nm using a spectrophotometer. Tocopherol was used as a standard [24].

#### 2.9.4. In Vitro Lipoxygenase (LOX) Inhibition Assay

The lipoxygenase inhibitory activity of *I. dipyrena* crude extract and fractions were also determined using standard method. About 1 mL sodium borate buffer (0.1 M, pH 8.8) was mixed with 10 mL of soybean LOX (8000 U/mL), which were then incubated at room temperature for 10 min with 10 mL of plant extract samples (31.25–1000 g/mL). The reaction was started by adding 10 mL of linoleic acid as a substrate to the mixture (10 mM). For every minute, the absorbance was measured at 234 nm of the resulting mixture for 6 min. The LOX inhibition was then calculated, and the inhibitory concentration (IC_50_) was determined. A positive control, indomethacin, was used [25].

### 2.10. Statistical Analysis

The obtained data were represented as mean ± SEM. For statistical analysis, one-way ANOVA followed by the Dunnett’s test was carried out using GraphPad Prism 5 version 5.01 (GraphPad Prism Software, Inc., San Diego, CA, USA). The results were claimed significant when the *p*-value was less than 0.05. 

## 3. Results

### 3.1. Macroscopic Evaluations

Morphological features of bark, stem, petiole, and leaf adaxial and abaxial surfaces are presented in Table 1. Height of the plant was assessed randomly, and the mean data of 10 plants were recorded. 

Upon examination of the *I. dipyrena* plant, the leaf color was found to be dark green and glassy on the adaxial side, while the abaxial side of leaf found to be light green in color. The shape of the leaf appeared to be lanceolate serrate. The leaves on the plant were alternate. The leaves of this plant are aromatic in nature upon bruising/crushing in hands, while their taste is bitter. The stem is angular in shape with dark green color. The average height of the plant was found to be 13.09 ± 1.01 m. The average length of leaf was 12.64 ± 1.62 cm with 6.03 ± 0.19-mm width. The length of lamina was 12.04 ± 0.91 with width of 4.16 ± 0.39 cm. All these parameters were helpful in the authentication of the plant.

### 3.2. Anatomical Characteristics of I. dipyrena

The anatomical characteristics of *I. dipyrena* were assessed for leaf, midrib, petiole, stem bark, and root bark. The recorded observations are described below.

#### 3.2.1. Inner Morphological Characteristics of the Leaf

**Figure 2:**
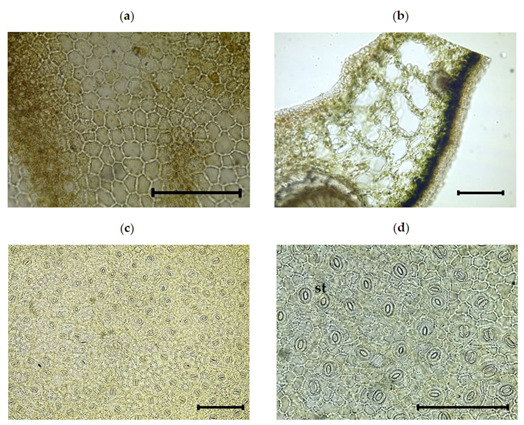
Photomicroscopic data of the leaf surface of *I. dipyrena*. (**a**) Adaxial (upper); (**b**) cross sectioned of leaf; and (**c**) and (**d**) abaxial (lower) surface. The black bars represent 100 µm. st, stomata.

The foliar epidermis of *I. dipyrena* characteristics are listed in Table 2, whereas Figure 2a,b shows its photographs. The protective layer called the cuticle is clear around the leaf, whereas the adaxial and abaxial epidermis were found in prominent arrangement. The adaxial and abaxial epidermis are protected by the cuticle. There are no trichomes on both surfaces of the leaves, confirming that leaves are glabrous. The adaxial epidermal cells consists of smooth anticlinal walls, while the abaxial epidermal cells possess slightly undulating walls. Stomata are only confined to the abaxial surface. Such leaves are called hypostomatic. The stomata, on the other hand, are of the anomocytic type, with an average stomatal index of 9.0 ± 1. Stomata are found on the same level as the surrounding epidermal cells, which means that they are connected. 

#### 3.2.2. Inner Morphological Features of the Midrib

**Figure 3:**
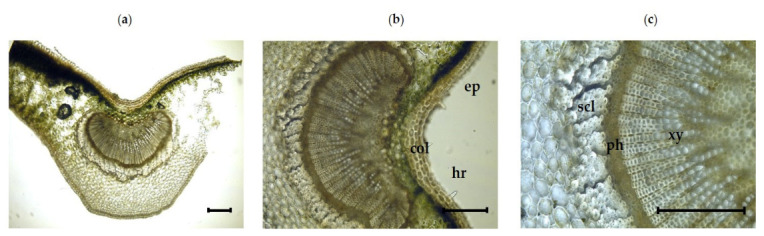
Photomicroscopic data of the *I. dipyrena* midrib: (**a**) transverse section of the midrib; (**b**) and (**c**) transverse section of a U-shaped vascular bundle located at the center of the midrib. The black bars represent 100 µm. ep, epidermis; col, collenchyma cells; hr, hair; xy, xylem; ph, phloem.

In a transverse section across the midrib area of the leaf, the salient characteristics of the midrib in the lower portion of the leaf were seen (Table 3; Figure 3a–c). The epidermis is made up of a single layer of cells that are covered by a thin cuticle. The midrib is largely made up of ground tissue with central vascular bundles and is mostly comprised of parenchyma cells. Angular collenchyma in clusters were found along with the lower and upper epidermis. Radial vessel layers were observed in the center of the midrib. In the midrib, the vascular system, comprised of a single, U-shaped collateral vascular bundle, was observed. The xylem on the adaxial side and phloem on the abaxial side were also identified in U-shaped collateral vascular bundle (Figure 3a–c). The phloem comprises of phloem parenchyma, while the xylem consists of vessels. An interrupted sheath of fibers was observed in groups of two to five or more, which abuts surrounding the base of the phloem. The fibers were round, oval, or polygonal in cross section. (Figure 3c). Non-glandular trichomes were found. In addition, druses (calcium oxalate) were found very rarely in the ground parenchyma.

#### 3.2.3. Inner Morphological Features of the Petiole

**Figure 4:**
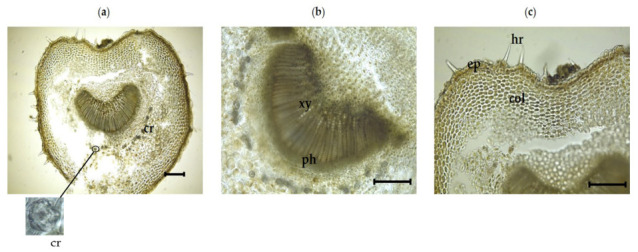
Photomicroscopic data of the *I. dypyrena* petiole: (**a**) petiole transverse section; (**b**) and (**c**) transverse section of a central vascular bundle of the petiole. The black bars represent 100 µm. cr, druse; ep, epidermis; col, collenchyma cells; hr, hair; xy, xylem; ph, phloem.

In a transverse section across the petiole area of the leaf, the distinct features of the midrib in the lower portion of the leaf were seen clearly (Table 4) (Figure 4a–c). Non-glandular unicellular hairs were found around the petiole. The petiole is stout, short, and about 2–3-mm long. The epidermis has similar characteristics as the midrib. Non-glandular trichrome were observed around the petiole. The epidermal layer is followed by up to twelve (adaxial) and eight (abaxial) layers of angular collenchyma. Parenchymatous ground tissue makes up a large component of the petiole. At the center of the petiole lies a large, U-shaped vascular bundle comparable to that of the midrib. The calcium oxalate crystals, such as druse, were observed around the central vascular bundle. 

#### 3.2.4. Inner Morphological Features of the Stem Bark

**Figure 5:**
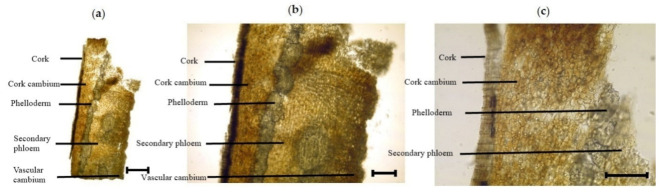
Photomicroscopic data of the *I. dipyrena* stem bark: (**a**), (**b**), and (**c**) are the transverse section of the stem bark. The black bars mean 100 µm.

The cross section of stem bark of *I. dipyrena* shows different types of tissues (Figure 5). The outermost layer is composed of dead cell called cork that protects the internal layers. Cork is followed by a layer called cork cambium. It is the meristematic tissue that continuously forms new cells for the formation of cork on outside and phelloderm on inside. The latter is a compact layer of cells located just beneath the cork cambium. It is followed by a thicker but relatively loose secondary phloem tissue, which is responsible for the girth of stem during secondary growth. The layer that is responsible for the formation of secondary phloem is located beneath and called vascular cambium. It is the main growth layer of the stem. 

#### 3.2.5. Inner Morphological Characteristics of the Root Bark

**Figure 6:**
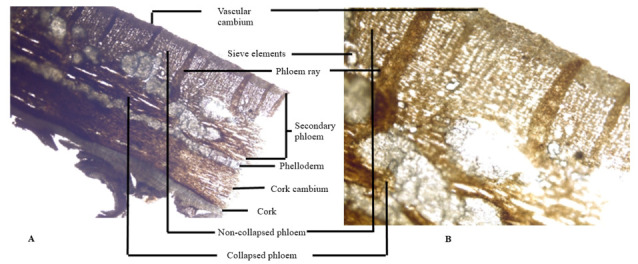
Photomicroscopic data of the *I. dipyrena* root bark. (**A**) and (**B**) are the transverse section of the root bark.

The cross section of root bark of *I. dipyrena* shows a complex structure of different tissues (Figure 6). The outermost layer is cork just like the stem bark, which is followed by a cork cambium. The phelloderm of the root is narrow and compact. The secondary phloem is clearly differentiable in loose sieve elements and elongated horizontally arranged phloem rays. The vascular cambium is located just beneath the secondary phloem. The overall cross section of the root seems to have implications in identification of the species due to the presence of horizontally arranged elongated phloem rays. 

### 3.3. Quantitative Phytochemical Analysis of Alkaloids, Flavonoids, Saponins, and Terpenoids

*I. dipyrena* was assessed for the presence phytochemicals, and results of quantitative phytochemical analysis of the crude extract and fractions are presented in Table 5.

### 3.4. Determination of Mineral Contents

*I. dipyrena* was also analyzed for various minerals, i.e., calcium (Ca^2^), potassium (K), magnesium (Mg^2^), iron (Fe), manganese (Mn), and zinc (Zn). Mineral contents were detected using Atomic Absorption Spectrophotometer and flame photometer. The results are presented in Table 6 as follows: 

### 3.5. Phenolic Compounds Identification through HPLC-UV

**Figure 7:**
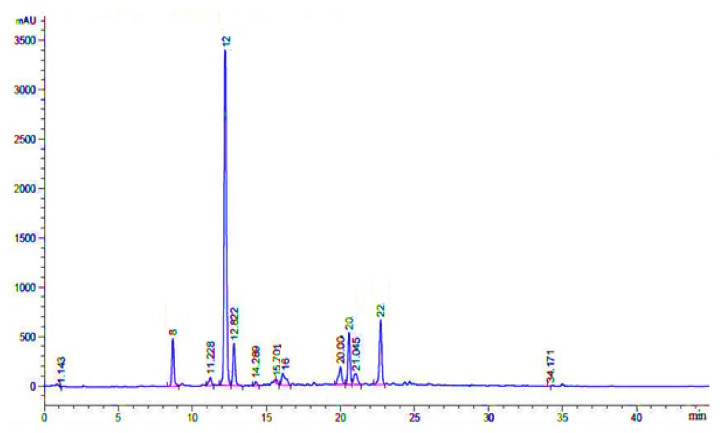
HPLC-UV chromatogram of *I. dipyrena* crude methanolic extract (recorded at 350 nm).

**Figure 8:**
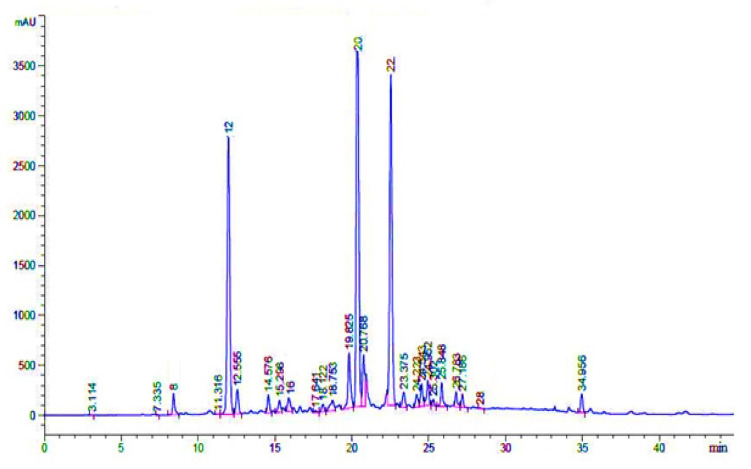
HPLC-UV chromatogram of *I. dipyrena* ethyl acetate extract (recorded at 350 nm).

**Figure 9:**
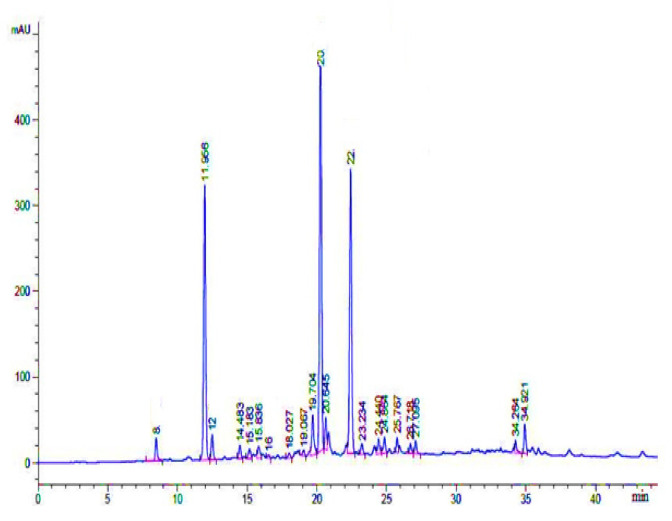
HPLC-UV chromatogram of *I. dipyrena* chloroform extract (recorded at 350 nm).

The possible compounds that were identified through HPL-UV analysis are presented in Table 7, while their typical chromatograms are shown in Figure 7, Figure 8 and Figure 9. Although there were many peaks related to different compounds, among them, few were identified. However, it should be noted that on the same retention time, there would be many organic compounds; therefore, the estimation made here is on probability basis, and thus, the word “possible” has added in front of them. In Crd-Id, the possible compounds were morin, epigallocatechin gallate, ellagic acid, rutin, and catechin hydrate (Figure 7), whereas in Et-Id, there were seven possible compounds: morin, malic acid, ellagic acid, epigallocatechin gallate, catechin hydrate, rutin, and pyrogallol (Figure 8); in Chl-Id, five possible phenolic compounds were epigallocatechin gallate, ellagic acid, morin, rutin, and catechin hydrate (Figure 9). Table 7 shows the concentrations of each of these compounds in a given extract along with peak position and retention times. 

### 3.6. Pharmacological Activities

#### 3.6.1. Antioxidant Activity

Table 8 shows the extracts’ and standard’s free-radical scavenging potential. The extracts showed concentration-dependent activity like that of the used standard.

Among the tested extracts, the most promising result was shown by Chl-Id, with IC_50_ value of 64.99, 69.15, and 268.52 µg/mL against DPHH, ABTS, and FRAP (Table 9). The ethyl acetate fraction was the second most active fraction, with IC_50_ values 72.57, 108.30, and 265.84 µg/mL against DPPH, ABTS, and FRAP, respectively.

#### 3.6.2. In Vitro Lipoxygenase Activity

Table 10 shows the in-vitro lipoxygenase activities of crude extract, fractions, and standard. The IC_50_ values of 75.99 and 106.11 µg/mL were recorded for chloroform and ethyl acetate extracts, whereas for standard, the value is 20.53 µg/mL. Again, a concentration-dependent response has been recorded as observed above.

## 4. Discussion

The authentication of plant material, particularly of medicinal plants, is critical when they are utilized as a medicinal source. Several methods for identification of plant products are available that are used to ensure quality and safety of the product. Such identification methods can assist in preventing mishaps at any point in the manufacturing process, from the raw material collection till finished product. Among these methods, anatomical studies are well-known in plant taxonomy and classification. Anatomical investigations are also useful in botanical quality control and pharmacognosy [26]. As a result, countless reports on plant anatomy are published on a frequent basis all around the world. However, thorough anatomical research for many taxa are still lacking. There are no anatomical or inner morphological details in the previously reported literature for *I. dipyrena*. Therefore, anatomical examination of *I. dipyrena* was performed using microscopic inspection.

The anatomy of *I. dipyrena* exhibits a number of interesting features. The leaves are hypostomatic and dorsiventral, with a single-layered epidermis and anomocytic stomata. The midrib and petioles have many of non-glandular trichomes. The U-shaped vascular bundle, which is large in size, is concentrated in the center of midrib. A shallow coating of fibers surrounds the phloem. A massive, U-shaped vascular bundle was also observed in the petiole. Druses of calcium oxalate crystals were also noticed. Druses are prevalent in the midrib and petiole’s ground tissue of petiole. In addition to anatomical study, DNA barcoding, a molecular technique usually used for the identification of plant species, should be used for more authentication of *I. dipyrena*.

Phytochemicals are naturally occurring secondary metabolites in plants and exhibit defensive, protective, and curative potential. Their regular intake as dietary sources may promote healthy life by protecting against various diseases [27]. It was confirmed from the preliminary phytochemical test that *I. dipyrena* contained phytoconstituents, such as glycosides, alkaloids, tannins, steroids, saponins, flavonoids, terpenoids, proteins, fats, and carbohydrates, which was consistent with the findings for other species of the genus *Ilex* [10,28]. *I. dipyrena* has a broad array of secondary metabolites, which are assumed to be involved in its diverse pharmacological potential.

HPLC, as a chemical fingerprinting approach, can be used to quickly determine the authenticity of plants and their botanical products. HPLC-UV analysis were used to confirm the phenolic or other chemical constituents found in the whole-plant extracts (Figure 7, Figure 8 and Figure 9). Several chemical constituents, such as epigallocatechin gallate, malic acid, catechin hydrate, rutin, morin, and pyrogallol, were found in *I. dipyrena* extracts. Through comparison of their HPLC chromatograms, significant difference in chemical profiles for whole-plant extracts of *I. dipyrena* plant samples were noticed as depicted in Figure 7, Figure 8 and Figure 9, suggesting that chemical contents changed significantly for different solvents. The ethyl acetate extract showed high contents of secondary metabolites but the least in chloroform extract [29].

Free radicals are extremely reactive and toxic substances that can lead to a wide range of health problems, including aging, diabetes, cancer, and atherosclerosis. They have also been linked to cardiovascular and liver diseases [30]. Antioxidants, particularly phenolic and flavonoids, have the capacity to scavenge free radicals, such as superoxide and hydroxyl radicals, effectively because of the presence of benzene rings in their structures [31]. To investigate the antioxidant profile of the *I. dipyrena* extracts, in vitro assays, such as scavenging of DPPH, ABTS, and FRAP free radical, were used. DPPH radical scavenging is considered a milestone in assessing the antioxidant potential of plant extracts. Among the tested samples, ethyl acetate was found to be a powerful scavenger of the tested free radical, whose activity was comparable with positive control. In addition, the contents of detected possible antioxidants in ethyl acetate were highest among the tested sample, which was consistent with the observed antioxidant activity. Overall, the crude extract and fractions exhibited promising antioxidant activity. However, the extent of free radical scavenging was different for DPPH, ABTS, and FRAP assays. This is due to the differences in mechanism of action between DPPH, ABTS, and FRAP assays. ABTS is based on hydrogen atom transfer (HAT) mechanism; FRAP is based on electron transfer (ET) antioxidant mechanism, whereas DPPH is a mixed assay (both HAT and ET). Here, the results showed that the HAT antioxidant mechanism is certainly the most prominent one. In addition, our results of the present study were in close agreement with reported studies where morin, quercetin, and rutin phytochemical were recognized as antioxidants in the extracts of *Ilex* species [29,32,33]. However, more research is needed in terms of isolating bioactive secondary metabolites and testing their toxicological effects in animal models to confirm the findings.

## 5. Conclusions

To the best of our knowledge, this is the first report on *I. dipyrena* anatomical, phytochemical, antioxidant, and antilipoxigenase potential. The research work uncovered notable traits in the morphology of *I. dipyrena* leaf, midrib, petiole, stem, and root barks that can aid in species taxonomy, identification, and quality control. Unicellular trichromes were found on midrib and petiole. The calcium oxalate druses were also observed in the midrib and petiole ground parenchyma. A number of phytochemicals were detected through HPLC analysis. The ethyl acetate extract showed high contents of secondary metabolites that were relatively low in chloroform extract. Furthermore, the chloroform and ethyl acetate fraction showed potent antioxidant activity as compared to other extracts. The findings of the study clearly suggest that *I. dipyrena* is an excellent source of bioactive compounds responsible for antioxidant and antilipoxigenase activities. These characteristics can be attributed to the presence of intrinsically active chemical constituents, such as flavonoids and saponins, which are present in the highest concentrations in *I. dipyrena*. However, further studies are required to isolate the pharmacologically active secondary metabolites responsible for observed biological potentials.

## Figures and Tables

**Table 1:** Organoleptic and macroscopic evaluation of *I. dipyrena*.

Sample	*Parameters/*Mean ± SEM
Leaf Color	Adaxial: dark green and glossy; Abaxial: light green
Leaf Pattern	Alternate
Leaf Shape	Lanceolate serrate
Stem color	Dark green, angular
Fruit Color	Shiny red
Odor	Aromatic
Taste	Bitter acidic
Height of Plant (m)	13.09 ± 1.01
Leaf length (cm)	12.64 ± 1.62
Petiole (mm)	L = 6.03 ± 0.19
Lamina (cm)	L = 12.04 ± 0.91, W = 4.16 ± 0.39

**Table 2:** Inner Morphological characteristics of the abaxial leaf surface of *I. dipyrena*.

Parameters	Surface	Range
Leaf stomatal frequency	Abaxial	145 ± 10
Stomatal index	Abaxial	9.0 ± 1
Length of stomata	Abaxial	27–31
Width of stomata	Abaxial	25–27
Stomatal type	Abaxial	Anomocytic
Palisade Tissue	-	2 layers
Palisade Tissue length	Adaxial	31–38
Palisade Tissue length	Abaxial	22–31

The values are reported as the mean standard deviation mean ± SD (*n* > 6).

**Table 3:** Inner morphological characteristics of the midrib of *I. dipyrena.*

Parameters	Surface	Range
Length of epidermal cells	Adaxial	14–16 µm
Width of epidermal cells	Adaxial	25–37 µm
Length of epidermal cells	Abaxial	9–11 µm
Width of epidermal cells	Abaxial	14–16 µm
Diameter of collenchyma cell	Adaxial	21–31 µm
Diameter of collenchyma cell	Abaxial	13–18 µm
Collenchyma cell layers	Adaxial	2–3
Collenchyma cell layers	Abaxial	4–5

**Table 4:** Inner morphological characteristics of the petiole of *I. dipyrena*.

Parameters	Surface	Range
Length of epidermal cells	Adaxial	14–15 µm
Width of epidermal cells	Adaxial	21–25 µm
Length of epidermal cells	Abaxial	14–17 µm
Width of epidermal cells	Abaxial	20–33 µm
Diameter of collenchyma cell	Adaxial	19–30 µm
Diameter of collenchyma cell	Abaxial	24–36 µm
Collenchyma cell layers	Adaxial	9–12
Collenchyma cell layers	Abaxial	6–8

**Table 5:** Non-spectrophotometric quantitative phytochemical analysis of crude extract and fractions of *I. dipyrena*.

Sample	Yield (%)			
	Alkaloids	Flavonoids	Saponins	Terpenoids
Crd-Id	2.86 ± 0.44	3.93 ± 0.83	5.15 ± 0.73	2.71 ± 0.49
Hex-Id	0.94 ± 0.34	1.31 ± 0.41	2.16 ± 0.65	1.03 ± 0.72
Chl-Id	1.69 ± 0.21	7.55 ± 0.47	4.22 ± 0.31	2.19 ± 0.45
Et-Id	3.14 ± 0.39	6.01 ± 0.63	3.71 ± 0.67	2.67 ± 0.76
But-Id	2.21 ± 0.71	3.28 ± 0.51	2.96 ± 0.71	1.69 ± 0.81
Aq-Id	1.75 ± 0.78	2.15 ± 0.61	2.03 ± 0.65	1.38 ± 0.56

All values are expressed as mean ± SEM, *n* = 3. Crd-Id, crude extract; Hex-Id, n-hexane fraction; Chl-Id, chloroform fraction; Et-Id, ethyl acetate fraction; But-Id, butanol fraction; Aq-Id, aqueous fraction.

**Table 6:** Mineral content of *I. dipyrena*.

Specimen	Mineral (mg/100 g Dry Weight)					
	Potassium	Calcium	Magnesium	Iron	Manganese	Zinc
*I. dipyrena*	297 ± 2.79	31.03 ± 1.01	34.68 ± 1.97	0.20 ± 0.04	0.92 ± 0.07	0.14 ± 0.01

Results are taken as mean ± SEM, *n* = 3.

**Table 7:** Identification and quantification of phenolic phytochemical compounds in *I. dipyrena*.

Extract	No. of Peak	Retention Time (min)	Detected Possible Phenolic Compounds	Sample Peak Area	Standard Peak Area	Concentration (µg/mL)
Crd-Id	1	8.00	Epigallocatechin gallate	4784.77	7261.47	6.58
	2	12.00	Morin	3.814	20.0	1.907
	3	16.00	Ellagic acid	2260.81	319.24	70.81
	4	20.00	Catechin hydrate	2552.98	78.0	327.31
	5	22.00	Rutin	7122.74	2241.2	31.78
Et-Id	1	3.10	Malic acid	19.126	40.32	4.74
	2	8.00	Epigallocatechin gallate	2191.81	7261.47	3.68
	3	12.00	Morin	3.317	20.0	1.65
	4	16.00	Ellagic acid	1634.55	319.24	51.20
	5	20.00	Catechin hydrate	4.59	78.0	0.58
	6	22.00	Rutin	3.659	2241.2	0.016
	7	28.00	Pyrogallol	64.97	1.014	640.72
Chl-Id	1	8.00	Epigallocatechin gallate	274.08	7261.47	0.37
	2	12.00	Morin	271.35	20.0	135.67
	3	16.00	Ellagic acid	41.81	319.24	1.309
	4	20.00	Catechin hydrate	4546.75	78.0	582.91
	5	22.00	Rutin	3435.11	2241.2	15.32

Standard peak area is the area under a given peak of external standard, whereas sample peak area is the suspected compound area in sample chromatogram.

**Table 8:** Antioxidant activity of crude extract and different fractions.

Sample	Conc. (µg/mL)	%Inhibition		
		DPPH	ABTS	FRAP
Crd-Id	1000	74.30 ± 0.48	76.15 ± 0.59	57.31 ± 0.65
	500	71.26 ± 0.51	69.23 ± 0.69	38.61 ± 0.67
	250	65.40 ± 0.61	63.09 ± 0.61	35.05 ± 0.66
	125	49.58 ± 0.73	47.92 ± 0.67	32.81 ± 0.70
	62.5	39.10 ± 0.71	40.25 ± 0.57	29.15 ± 0.57
	31.25	33.52 ± 0.65	35.80 ± 0.63	26.27 ± 0.62
Hex-Id	1000	62.84 ± 0.63	62.73 ± 0.69	44.09 ± 0.61
	500	60.17 ± 0.67	57.98 ± 0.60	33.13 ± 0.56
	250	47.89 ± 0.59	46.92 ± 0.67	30.01 ± 0.61
	125	41.08 ± 0.61	39.20 ± 0.56	27.14 ± 0.69
	62.5	30.14 ± 0.59	33.29 ± 0.49	25.10 ± 0.52
	31.25	31.20 ± 0.60	30.17 ± 0.65	21.72 ± 0.59
Chl-Id	1000	75.79 ± 0.81	77.83 ± 0.61	61.41 ± 0.71
	500	70.51 ± 0.76	72.50 ± 0.66	58.71 ± 0.63
	250	65.05 ± 0.67	62.79 ± 0.71	46.55 ± 0.61
	125	56.41 ± 0.61	60.12 ± 0.61	40.12 ± 0.66
	62.5	48.08 ± 0.55	45.19 ± 0.52	29.72 ± 0.57
	31.25	41.05 ± 0.61	39.26 ± 0.63	29.01 ± 0.71
Et-Id	1000	78.09 ± 0.58	74.23 ± 0.66	60.13 ± 0.60
	500	70.31 ± 0.70	69.08 ± 0.73	59.71 ± 0.58
	250	63.81 ± 0.61	65.21 ± 0.66	47.02 ± 0.67
	125	61.09 ± 0.65	57.71 ± 0.65	39.72 ± 0.62
	62.5	43.06 ± 0.59	39.09 ± 0.59	28.41 ± 0.70
	31.25	38.16 ± 0.64	37.74 ± 0.67	27.59 ± 0.59
Bt-Id	1000	75.80 ± 0.73	59.40 ± 0.61	45.72 ± 0.55
	500	70.15 ± 0.71	55.19 ± 0.67	35.34 ± 0.61
	250	66.16 ± 0.59	41.79 ± 0.59	33.60 ± 0.63
	125	50.76 ± 0.56	37.28 ± 0.60	30.41 ± 0.59
	62.5	40.16 ± 0.61	33.78 ± 0.68	27.62 ± 0.60
	31.25	38.90 ± 0.59	31.91 ± 0.63	24.89 ± 0.63
Aq-Id	1000	75.81 ± 0.79	60.98 ± 0.71	40.14 ± 0.70
	500	68.92 ± 0.71	57.01 ± 0.56	32.19 ± 0.63
	250	62.14 ± 0.69	40.99 ± 0.67	29.67 ± 0.59
	125	47.63 ± 0.58	39.43 ± 0.63	27.78 ± 0.61
	62.5	39.12 ± 0.64	35.80 ± 0.61	24.97 ± 0.70
	31.25	34.23 ± 0.61	33.74 ± 0.59	22.49 ± 0.67
Tocopherol	1000	98.07 ± 0.57	95.11 ± 0.65	99.65 ± 0.68
	500	96.89 ± 0.61	94.34 ± 0.59	98.38 ± 0.63
	250	96.12 ± 0.67	93.26 ± 0.60	96.59 ± 0.61
	125	95.18 ± 0.70	92.09 ± 0.67	95.42 ± 0.56
	62.5	93.03 ± 0.61	91.01 ± 0.59	94.11 ± 0.57
	31.25	90.17 ± 0.71	89.72 ± 0.63	92.19 ± 0.61

Each value is represented as Mean ± SEM (*n* = 3).

**Table 9:** IC_50_ values of crude extract and fractions.

Sample	IC_50_ (µg/mL)		
	DPPH	ABTS	FRAP
Crd-Id	126.05	130.42	877.19
Hex-Id	261.01	266.41	>1000
Chl-Id	64.99	69.15	268.52
Et-Id	72.57	108.30	265.84
Bt-Id	123.12	452.98	>1000
Aq-Id	131.21	438.51	>1000
Tocopherol	17.13	17.41	16.94

**Table 10:** Percent inhibition of lipoxygenase activity with IC_50_ values.

Sample	Conc. (µg/mL)	%Inhibition	IC_50_ (µg/mL)
Crd-Id	1000	76.03 ± 0.61	133.83
	500	69.11 ± 0.59	
	250	61.45 ± 0.67	
	125	46.70 ± 0.71	
	62.5	40.03 ± 0.61	
	31.25	36.29 ± 0.58	
Hex-Id	1000	64.34 ± 0.64	266.46
	500	60.17 ± 0.61	
	250	46.91 ± 0.59	
	125	40.03 ± 0.60	
	62.5	30.38 ± 0.69	
	31.25	28.93 ± 0.52	
Chl-Id	1000	79.13 ± 0.69	75.99
	500	71.61 ± 0.63	
	250	64.49 ± 0.61	
	125	60.12 ± 0.53	
	62.5	45.12 ± 0.64	
	31.25	40.72 ± 0.71	
Et-Id	1000	74.13 ± 0.66	106.11
	500	70.21 ± 0.59	
	250	66.09 ± 0.60	
	125	58.90 ± 0.61	
	62.5	40.65 ± 0.63	
	31.25	38.62 ± 0.58	
Bt-Id	1000	64.30 ± 0.69	228.89
	500	61.75 ± 0.61	
	250	54.61 ± 0.59	
	125	41.27 ± 0.60	
	62.5	31.89 ± 0.63	
	31.25	30.28 ± 0.58	
Aq-Id	1000	63.32 ± 0.54	250.15
	500	56.80 ± 0.59	
	250	49.97 ± 0.61	
	125	40.21 ± 0.65	
	62.5	34.90 ± 0.58	
	31.25	31.20 ± 0.63	
Indomethacin	1000	93.21 ± 0.65	20.53
	500	92.17 ± 0.59	
	250	90.10 ± 0.70	
	125	87.06 ± 0.58	
	62.5	83.41 ± 0.69	
	31.25	76.09 ± 0.64	

Each value is represented as Mean ± SEM (*n* = 3).

## Data Availability

Data are contained within the article.

## References

[1] Shah M., Murad W., Rehman N.U., Halim S.A., Ahmed M., Rehman H., Zahoor M., Mubin S., Khan A., Nassan M.A. (2021). Biomedical Applications of *Scutellaria edelbergii* Rech. f.: In Vitro and In Vivo Approach. Molecules.

[2] Ul Bari W., Ur Rehman N., Khan A., Halim S.A., Yuan Y., Blaskovich M.A.T., Ziora Z.M., Zahoor M., Naz S., Ullah R. (2021). Bio-Potency and Molecular Docking Studies of Isolated Compounds from *Grewia optiva* J.R. Drumm. ex Burret. Molecules.

[3] Zahoor M., Khan I., Zeb A., Sahibzada M.U.K., Naz S., Bari W.U., Kamran A.W. (2021). Pharmacological evaluation and in-silico modeling study of compounds isolated from *Ziziphus oxyphylla*. Heliyon.

[4] Nazir N., Zahoor M., Uddin F., Nisar M. (2021). Chemical composition, in vitro antioxidant, anticholinesterase, and antidiabetic potential of essential oil of *Elaeagnus umbellata* Thunb. BMC Complement. Med. Ther..

[5] Khalil A., Tazeddinova D. (2020). The upshot of Polyphenolic compounds on immunity amid COVID-19 pandemic and other emerging communicable diseases: An appraisal. Nat. Prod. Bioprospect..

[6] Gaur R. (1999). Flora of the District Garhwal, North West Himalaya.

[7] Zuo W.-J., Dai H.-F., Chen J., Chen H.-Q., Zhao Y.-X., Mei W.-L., Li X., Wang J.-H. (2011). Triterpenes and Triterpenoid Saponins from the Leaves of *Ilex kudincha*. Planta Med..

[8] Vickers N.J. (2017). Animal communication: When I’ m calling you, will you answer too?. Curr. Biol..

[9] Wang S., Kirillova K., Lehto X. (2017). Travelers’ food experience sharing on social network sites. J. Travel Tour. Mark..

[10] Hao D., Gu X., Xiao P., Liang Z., Xu L., Peng Y. (2013). Research progress in the phytochemistry and biology of *Ilex* pharmaceutical resources. Acta Pharm. Sin. B.

[11] Kim J.Y., Lee H.K., Seong Y.H. (2019). Anti-nociceptive and anti-inflammatory properties of *Ilex latifolia* and its active component, 3,5-di-caffeoyl quinic acid methyl ester. Nat. Prod. Sci..

[12] Kothiyal S.K., Sati S.C., Rawat M.S.M., Sati M.D., Semwal D.K., Semwal R.B., Sharma A., Rawat B., Kumar A. (2012). Chemical constituents and biological significance of the genus *Ilex* (Aquifoliaceae). Nat. Prod. J..

[13] Nowacki L.C., Stechman-Neto J., Schiefer E.M., Santos A.F., Stinghen A.E., Sassaki G.L., De Souza L.M., Cristoff K.E., De Souza W.M. (2021). *Ilex* paraguariensis extract as drugs alternative for pain. Acta Pharm..

[14] De Carvalho E.F., de Oliveira S.K., Nardi V.K., Gelinski T.C., Bortoluzzi M.C., Maraschin M., Nardi G.M. (2016). *Ilex paraguariensis* promotes orofacial pain relief after formalin injection: Involvement of noradrenergic pathway. Pharmacogn. Res..

[15] Kothiyal S.K., Semwal D.K., Badoni R., Rawat U. (2010). GC-MS analysis of fatty acids and the antimicrobial activity of *Ilex dipyrena* Wallich leaves. Asian J. Tradit. Med..

[16] Ali A., Nasir A., Shah S.W.A., Khalil A.A.K., Ahn M.-J., Shah S.M.M., Subhan F., Faheem M., Sajjad W., Shoaib M. (2021). Evaluation of antinociceptive activity of *Ilex dipyrena* Wall. in mice. BMC Complement. Med. Ther..

[17] Ali A., Khalil A.A.K., Khuda F., Nazir N., Ullah R., Bari A., Haider A., Jamal S.B., Ahmad S., Khan Z. (2021). Phytochemical and Biological Screening of Leaf, Bark and Fruit Extracts from *Ilex dipyrena* Wall. Life.

[18] WHO (1998). Quality Control Methods for Medicinal Plant Materials.

[19] Khalil A.A.K., Akter K.-M., Kim H.-J., Park W.S., Kang D.-M., Koo K.A., Ahn M.-J. (2020). Comparative inner morphological and chemical studies on *Reynoutria* species in Korea. Plants.

[20] Batool R., Khan M.R., Sajid M., Ali S., Zahra Z. (2019). Estimation of phytochemical constituents and in vitro antioxidant potencies of *Brachychiton populneus* (Schott & Endl.) R.Br.. BMC Chem..

[21] El-Mogy M.M., Mahmoud A.W.M., El-Sawy M.B., Parmar A. (2019). Pre-harvest foliar application of mineral nutrients to retard chlorophyll degradation and preserve bio-active compounds in broccoli. Agronomy.

[22] Nazir N., Khalil A.A.K., Nisar M., Zahoor M., Ahmad S. (2020). HPLC-UV characterization, anticholinesterase, and free radical-scavenging activities of *Rosa moschata* Herrm. leaves and fruits methanolic extracts. Rev. Bras. Bot..

[23] Khuda F., Haq Z.U., Ilahi I., Ullah R., Khan A., Fouad H., Khalil A.A.K., Ullah Z., Sahibzada M.U.K., Shah Y. (2021). Synthesis of gold nanoparticles using *Sambucus wightiana* extract and investigation of its antimicrobial, anti-inflammatory, antioxidant and analgesic activities. Arab. J. Chem..

[24] Asraoui F., Kounnoun A., Cadi H.E., Cacciola F., Majdoub Y.O.E., Alibrando F., Mandolfino F., Dugo P., Mondello L., Louajri A. (2021). Phytochemical Investigation and Antioxidant Activity of *Globularia alypum* L.. Molecules.

[25] Naz R., Roberts T.H., Bano A., Nosheen A., Yasmin H., Hassan M.N., Keyani R., Ullah S., Khan W., Anwar Z. (2020). GC-MS analysis, antimicrobial, antioxidant, antilipoxygenase and cytotoxic activities of Jacaranda mimosifolia methanol leaf extracts and fractions. PLoS ONE.

[26] Akter K.-M., Park W.S., Kim H.-J., Khalil A.A.K., Ahn M.-J. (2020). Comparative Studies of Fraxinus Species from Korea Using Microscopic Characterization, Phytochemical Analysis, and Anti-Lipase Enzyme Activity. Plants.

[27] Ahmed M., Ji M., Sikandar A., Iram A., Qin P., Zhu H., Javeed A., Shafi J., Iqbal Z., Farid Iqbal M. (2019). Phytochemical Analysis, Biochemical and Mineral Composition and GC-MS Profiling of Methanolic Extract of Chinese Arrowhead *Sagittaria trifolia* L. from Northeast China. Molecules.

[28] Lin L.-P., Kong X., Chen L., Chen L. (2019). Chemical constituents from the roots of cultivated *Ilex pubescens*. Biochem. Syst. Ecol..

[29] Ramirez-Mares M.V., Chandra S., De Mejia E.G. (2004). In vitro chemopreventive activity of Camellia sinensis, *Ilex paraguariensis* and *Ardisia compressa* tea extracts and selected polyphenols. Mutat. Res. Fundam. Mol. Mech. Mutagenesis.

[30] Alzandi A.A., Taher E.A., Al-Sagheer N.A., Al-Khulaidi A.W., Azizi M., Naguib D.M. (2021). Phytochemical components, antioxidant and anticancer activity of 18 major medicinal plants in Albaha region, Saudi Arabia. Biocatal. Agric. Biotechnol..

[31] Njoya E.M. (2021). Medicinal plants, antioxidant potential, and cancer. Cancer.

[32] Liu L., Sun Y., Laura T., Liang X., Ye H., Zeng X. (2009). Determination of polyphenolic content and antioxidant activity of kudingcha made from *Ilex kudingcha* CJ Tseng. Food Chem..

[33] Filip R., Lotito S.B., Ferraro G., Fraga C.G. (2000). Antioxidant activity of *Ilex paraguariensis* and related species. Nutr. Res..

